# Genetic polymorphism of the N-terminal region in circumsporozoite surface protein of *Plasmodium falciparum* field isolates from Sudan

**DOI:** 10.1186/s12936-019-2970-0

**Published:** 2019-10-01

**Authors:** Nouh S. Mohamed, Musab M. Ali Albsheer, Hanadi Abdelbagi, Emanuel E. Siddig, Mona A. Mohamed, Abdallah E. Ahmed, Rihab Ali Omer, Mohamed S. Muneer, Ayman Ahmed, Hussam A. Osman, Mohamed S. Ali, Ibrahim M. Eisa, Mohamed M. Elbasheir

**Affiliations:** 1 Department of Parasitology and Medical Entomology, Faculty of Medical Laboratory Sciences, Nile College, Khartoum, Sudan; 2 Department of Parasitology and Medical Entomology, Faculty of Medical Laboratory Sciences, Sinnar University, Sinnar, Sudan; 3 Department of Molecular Biology, Institute of Zoology, University of Hohenheim, Stuttgart, Germany; 4 Department of Parasitology and Medical Entomology, East Nile College, Khartoum, Sudan; 5 Biotechnology Research Laboratory, School of Pharmacy, Ahfad University for Women, Omdurman, Sudan; 6 Unit of Applied Medical Sciences, Faculty of Medical Laboratory Sciences, University of Khartoum, Khartoum, Sudan; 7 Mycetoma Research Center, University of Khartoum, Khartoum, Sudan; 8 Department of Molecular Biology, Institute of Parasitology, University of Leipzig, Leipzig, Germany; 9 Department of Neurology, Mayo Clinic, Jacksonville, FL USA; 10 Department of Neurosurgery, Mayo Clinic, Jacksonville, FL USA; 11 Department of Internal Medicine, Faculty of Medicine, University of Khartoum, Khartoum, Sudan; 12 Department of Parasitology and Medical Entomology, Institute of Endemic Diseases, University of Khartoum, Khartoum, Sudan; 13 World Reference Center for Emerging Viruses and Arboviruses, University of Texas Medical Branch, Galveston, TX USA; 14 Faculty of Medicine, Neelain University, Khartoum, Sudan; 15 Department of Parasitology and Medical Entomology, Faculty of Medical Laboratory Sciences, Alzaiem Alazhari University, Khartoum, Sudan

**Keywords:** *Plasmodium falciparum*, Circumsporozoite protein, N-terminal region, Genetic polymorphism, Sudan

## Abstract

**Background:**

Malaria caused by *Plasmodium falciparum* parasite is still known to be one of the most significant public health problems in sub-Saharan Africa. Genetic diversity of the Sudanese *P. falciparum* based on the diversity in the circumsporozoite surface protein (PfCSP) has not been previously studied. Therefore, this study aimed to investigate the genetic diversity of the N-terminal region of the *pfcsp* gene.

**Methods:**

A cross-sectional molecular study was conducted; 50 blood samples have been analysed from different regions in Sudan. Patients were recruited from the health facilities of Khartoum, New Halfa, Red Sea, White Nile, Al Qadarif, Gezira, River Nile, and Ad Damazin during malaria transmission seasons between June to October and December to February 2017–2018. Microscopic and nested PCR was performed for detection of *P. falciparum*. Merozoite surface protein-1 was performed to differentiate single and multiple clonal infections. The N-terminal of the *pfcsp* gene has been sequenced using PCR-Sanger dideoxy method and analysed to sequences polymorphism including the numbers of haplotypes (H), segregating sites (S), haplotypes diversity (Hd) and the average number of nucleotide differences between two sequences (Pi) were obtained using the software DnaSP v5.10. As well as neutrality testing, Tajima’s D test, Fu and Li’s D and F statistics.

**Results:**

PCR amplification resulted in 1200 bp of the *pfcsp* gene. Only 21 PCR products were successfully sequenced while 29 were presenting multiple clonal *P. falciparum* parasite were not sequenced. The analysis of the N-terminal region of the PfCSP amino acids sequence compared to the reference strains showed five different haplotypes. H1 consisted of 3D7, NF54, HB3 and 13 isolates of the Sudanese *pfcsp*. H2 comprised of 7G8, Dd2, MAD20, RO33, Wellcome strain, and 5 isolates of the Sudanese *pfcsp*. H3, H4, and H5 were found in 3 distinct isolates. Hd was 0.594 ± 0.065, and S was 12. The most common polymorphic site was A98G; other sites were D82Y, N83H, N83M, K85L, L86F, R87L, R87F, and A98S. Fu and Li’s D* test value was − 2.70818, Fu and Li’s F* test value was − 2.83907, indicating a role of negative balancing selection in the *pfcsp* N-terminal region. Analysis with the global *pfcsp* N-terminal regions showed the presence of 13 haplotypes. Haplotypes frequencies were 79.4%, 17.0%, 1.6% and 1.0% for H1, H2, H3 and H4, respectively. Remaining haplotypes frequency was 0.1% for each. Hd was 0.340 ± 0.017 with a Pi of 0.00485, S was 18 sites, and Pi was 0.00030. Amino acid polymorphisms identified in the N-terminal region of global *pfcsp* were present at eight positions (D82Y, N83H/M, K85L/T/N, L86F, R87L/F, A98G/V/S, D99G, and G100D).

**Conclusions:**

Sudanese *pfcsp* N-terminal region was well-conserved with only a few polymorphic sites. Geographical distribution of genetic diversity showed high similarity to the African isolates, and this will help and contribute in the deployment of RTS,S, a PfCSP-based vaccine, in Sudan.

## Background

Malaria caused by *Plasmodium falciparum* parasite is still known to be one of the most significant public health problems in Africa [1]. In 2017, the global morbidity and mortality rate of the disease reached 216 million cases and a total of 450,000 deaths [1]. The infection is caused by the bite of infected female *Anopheles* mosquito, which injects the sporozoite, the infective stage of the parasite [2].

**Fig. 1:**
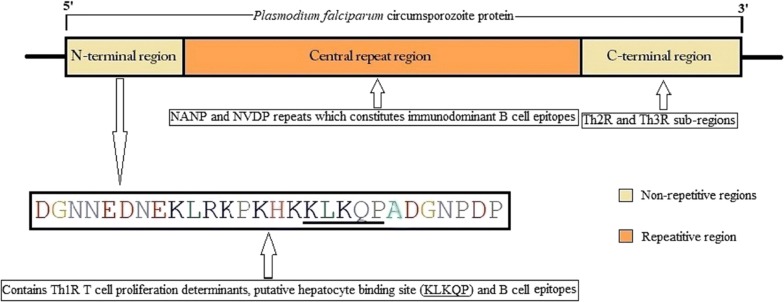
A schematic representation of the *pfcsp* gene showing the N-terminal region described in this study; DGNNEDNEKLRKPKHKKLKQPADGNPDP (underlined KLKQP motif responsible for the sporozoite entry into hepatocytes). In the central repeat region NANP (N, asparagine; A, alanine and P, proline) and NVDP (N, asparagine; V, valine; D, aspartic acid and P, proline) repeats. C-terminal region contains Th2R and Th3R epitopes

In Sudan, malaria continues to spread despite the efforts of the National Malaria Control Programme (NMCP). Many studies in Sudan have focused on addressing the situation of malaria treatment efficacy [3–5], while others focused on reporting the genetic diversity and the genetic makeup of the parasite itself [6–10]. RTS,S, which is the most advanced malaria vaccine to be implemented in most African countries, has shown a remarkable reduction of falciparum malaria episodes in children [11–13]. Many studies worldwide focused on addressing the genetic diversity of the local *P. falciparum* strains in order to develop an effective malaria vaccine [14–16]. The RTS,S malaria vaccine is based on the circumsporozoite protein of *P. falciparum* (PfCSP). It is composed of a liposome-based adjuvant, and virus-like elements of hepatitis B virus surface antigen (HBsAg) joined to a portion of PfCSP, the main surface protein expressed at the surface of the sporozoites [17]. It is also known to have an essential role in the process of sporozoites entry into the human hepatic cells [18–20]. It has approximately 420 amino acids and a molecular weight of 58 kDa. The gene that encodes PfCSP is subdivided into two non-repetitive regions, the N-terminal region and the C-terminal region (5′ and 3′ ends), and a variable central region consisting of multiple repeats of four-residues long motifs [21–23]. A schematic representation of the *pfcsp* gene is described in Fig. 1. The N-terminal region encompasses KLKQP motif, which is vital in the entry inside the hepatocytes [19], while the C-terminal region composes of a polymorphic Th2R and Th3R sub-regions [24]. The polymorphism of these sub-regions is believed to be a result of natural selection related to host immunity [25–27].

Studies on the *P. falciparum* genome showed that geographical variation could result in strain variation [28, 29]. Many studies showed the presence of divergence that led to the reduction of the vaccine efficacy or in some cases to block the vaccine in preventing the infection. Also, the low polymorphic nature studied thoroughly on the N-terminal region of *pfcsp* gene has the potential for this region to be a prominent constituent of a *pfcsp*-based vaccine [14, 16]. In Sudan, no data are addressing the situation of the genetic diversity of the Sudanese PfCSP, which may affect the deployment of the RTS,S vaccine in terms of efficacy reduction. Therefore, studying the genetic diversity of *P. falciparum,* specifically on the N-terminal region of *pfcsp*, is crucial and will also provide an update of the genetic make-up of the *P. falciparum* parasites circulating in a specific region to help in producing regional vaccines. This may also direct researchers to design an optimal universal vaccine [16, 30]. This study aimed at investigating the genetic polymorphism of the Sudanese *P. falciparum* based on the N-terminal region of *pfcsp*.

## Methods

**Fig. 2:**
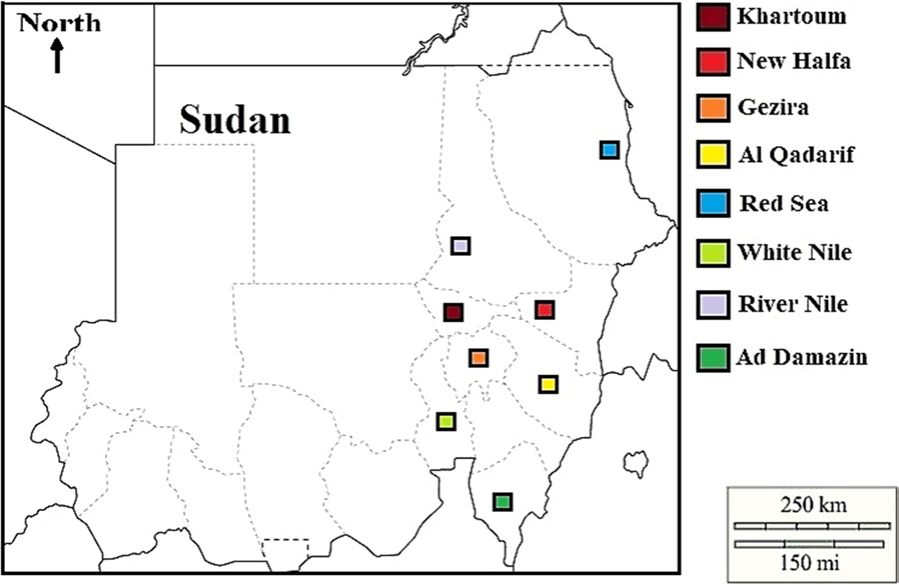
Map of Sudan showing sample collection sites. Solid squares indicate the locations of sample collection

A cross-sectional molecular study was carried out in different geographical areas in Sudan during the malaria transmission season in 2017–2018. These regions included Khartoum (15°55′N 32°53′E), New Halfa (15°35′N 35°39′E), Red Sea (19°35′N 35°37′E), White Nile (13°10′N 32°40′E), Al Qadarif (14°02′N 35°23′E), Gezira (14°30′N 33°30′E), River Nile (18°27′N 33°23′E) and Ad Damazin (11°46′N 34°21′E) (Fig. 2: Map of Sudan showing sample collection sites). The study areas are located in central, north, and east country. Based on malaria endemicity, Khartoum, Red Sea, White Nile, Gezira, and River Nile were considered as mesoendemic areas, while Al Qadarif, New Halfa and Ad Damazin are holo-endemic. In the studied areas, *P. falciparum* is the most common malaria parasite, responsible for 90% of malaria infections, while 10% are acknowledged to be caused by *Plasmodium vivax*. A total of 50 febrile patients in the representative health facilities of each area were recruited; a physician diagnoses those having malaria (positive microscopy, axillary temperature ≥ 37 °C). Before the beginning of treatment, 2 mL blood sample were collected in EDTA blood containers to prevent lysis. Informed consent from each patient or his/her legal guardians, in case of minors, were taken before sample collection. Demographical data, clinical data, and baseline information have been collected using the questionnaire interview.

Clinical phenotypes of malaria infection were assessed according to the WHO guidelines [31]. Microscopic examination was done using Giemsa-stained thick and thin blood film; two expert microscopist did the examination. The result was counted as positive when the reports of the two microscopists were positive. The collected blood samples were stored at 4 °C and transported to the Department of Molecular Biology at the National University Research Institute, Khartoum, for microscopic examination.

### Molecular detection and amplification of the *pfcsp* gene

The genomic DNA of *P. falciparum* isolates was extracted and purified from whole blood samples using the QIAamp DNA Blood Mini Kit (Qiagen Inc. Germany). The primers used for the detection of *P. falciparum* infection for further confirmation of the microscopic results were described previously by Snounou et al. [32]. Multiple clonal infections were determined using the primers published by Ntoumi et al. [33] to identify single and multiple allelic infections based on the Merozoite Surface Protein 1 (*msp1*). The amplification of the *pfcsp* was done according to Zeeshan et al. [14]; using the primers *pfcsp* F1: 5-TTAGCTATTTTATCTGTTTCTTC-3 and *pfcsp* R1: 5-TAAGGAACAAGAAGGATAATACC-3, followed by a nested PCR using the primers *pfcsp* F2 and *pfcsp* R2; 5-GAAATGAATTATTATGGGAAACAG-3 and 5-GAAGGATAATACCATTATTAATCC-3, respectively. The amplified DNA products were visualized using the agarose gel electrophoresis (BioMetra, Germany). 2% agarose gel in 1× TBE buffer stained with 3 µL Ethidium bromide (10 mg/mL). 5 µL of the PCR products were mixed with 3 µL of loading dye before loading into the gel wells. 100 base pair DNA marker was run with the sample in parallel wells. The gel was run for 60 min in 1× TBE buffer at 90 V. Finally, the gel was photographed using UV trans-illuminator (BioDoc-it, Germany). A duplicate of the amplified PfCSP PCR products was sequenced in the two directions using the primer *pfcsp* F3: 5-TGGGTCATTTGGCATATTGTG-3 by the Sanger dideoxy sequencing method using ABI3500 (Applied Biosystems SeqStudio, 3500 series) provided by Beijing Genomics Institute (BGI, China).

### Bioinformatics analysis

The C-terminal and the central region of the *pfcsp* gene were not sequenced. Therefore, only the N-terminal region was included in this study. Identity of amplified *pfcsp* products and percentages of similarity to *pfcsp* sequences available in the NCBI GenBank database was done using BLAST nucleotide algorithm (http://www.ncbi.nlm.nih.gov/Blast.cgi). For sequence diversity in comparison to the worldwide *pfcsp* sequences, all sequences deposited in the NCBI database that represent the N-terminal region of the *pfcsp* gene has been included in this analysis. The sequences were analysed for the identification of novel *P. falciparum* gene sequence polymorphism in the N-terminal region of the *pfcsp* reference strains, including 3D7 (XM_001351086), NF54 (M22982.1), HB3 (AB121018.1), 7G8 (AB121015.1), Dd2 (AB121017.1), MAD20 (AB121020.1), RO33 (AB121021.1) and Wellcome strain (M15505.1) using MEGA7 software. The construction of the phylogenetic tree was based on the maximum likelihood method. The model with the lowest BIC scores (Bayesian Information Criterion) was considered the best model to describe the nucleotides substitution patterns. Jukes and Cantor’s model was used for constructing the phylogenetic tree using MEGA7 software [34]. The deduced amino acids were translated from nucleotide sequences in order to investigate sequences diversity such as the numbers of haplotypes (H), segregating sites (S), haplotypes diversity (Hd) and the average number of nucleotide differences between two sequences (p) were obtained using the software DnaSP v5.10 [35]. For testing the neutrality of the N-terminal region of PfCSP, Tajima’s D test [36], Fu and Li’s D and F statistics analysis [37] were performed using DnaSP v5.10 to estimate the neutral theory of natural selection.

## Results

Descriptive, socio-demographic and clinical data of the recruited patients were presented in Additional file 1: Table S1. Nested PCR results for microscopic results confirmation were 100% sensitive and specific for the presence of *P. falciparum* parasite DNA. Also, results of *msp1* genotyping showed the presence of 21 single allelic infections and 29 multiple allelic infections. Nested PCR results and allelic frequency of MAD20, K1 and RO33 single and multiple allelic infections were also described in Additional file 1: Table S2. The amplified products obtained for the *pfcsp* were approximately 1200 bp in length as shown in Additional file 2: Figure S1. A total of 21 samples with mono-infection were successfully sequenced for the N-terminal region of the *pfcsp*, while the remaining 29 samples were not successfully sequenced due to the presence of multiple allelic *P. falciparum* infection.

### Sequence analysis of the Sudanese *pfcsp* N-terminal region

Identity of amplified *pfcsp* products and percentages of similarity to sequences available in the NCBI GenBank database using BLAST nucleotide algorithm showed an identity similarity to published *pfcsp* sequences with an identity ranged from 82.95 to 98.59% (Table 1).

**Table 1:** Study isolates similarity to the published sequences of PfCSP N-terminal region

Sequence no.	Study isolate location	Accession	Similarity with isolate	Isolate location	Percent identity (%)
Sequence 1	Khartoum	AB502803.1	Tz93-028	Tanzania	89.97
Sequence 2	Khartoum	AB502887.1	Gha1110-021	Ghana	94.80
Sequence 3	Al Qadarif	LR131340.1	HB3	Honduras	93.21
Sequence 4	Red Sea	AB502845.1	Tz98-119	Tanzania	93.82
Sequence 5	Red Sea	AB502845.1	Tz98-119	Tanzania	91.60
Sequence 6	White Nile	LR536676.1	7G8	Brazil	94.44
Sequence 7	White Nile	LR536676.1	7G8	Brazil	89.03
Sequence 8	New Halfa	AB502815.1	Tz93-060	Tanzania	94.69
Sequence 9	River Nile	AB502846.1	Tz03-025	Tanzania	84.19
Sequence 10	River Nile	AB116603.1	96M320-74	Vanuatu	88.00
Sequence 11	River Nile	AB503023.1	PNG828-140	Papua New Guinea	88.77
Sequence 12	New Halfa	MF350672.1	B32-4	Myanmar	92.97
Sequence 13	White Nile	AB827734.1	PFS96	Thailand	91.22
Sequence 14	Al Qadarif	LR536676.1	7G8	Brazil	94.34
Sequence 15	Ad Damazin	AB502838.1	Tz98-070	Tanzania	98.59
Sequence 16	Gezira	AB502815.1	Tz93-060	Tanzania	96.25
Sequence 17	Red Sea	AB502815.1	Tz93-060	Tanzania	97.24
Sequence 18	Gezira	MF350671.1	B7	Thailand	91.17
Sequence 19	Gezira	AB502843.1	Tz98-103	Tanzania	82.95
Sequence 20	Ad Damazin	AB502849.1	Tz03-038	Tanzania	93.45
Sequence 21	Ad Damazin	AB502849.1	Tz03-038	Tanzania	96.53

**Fig. 3:**
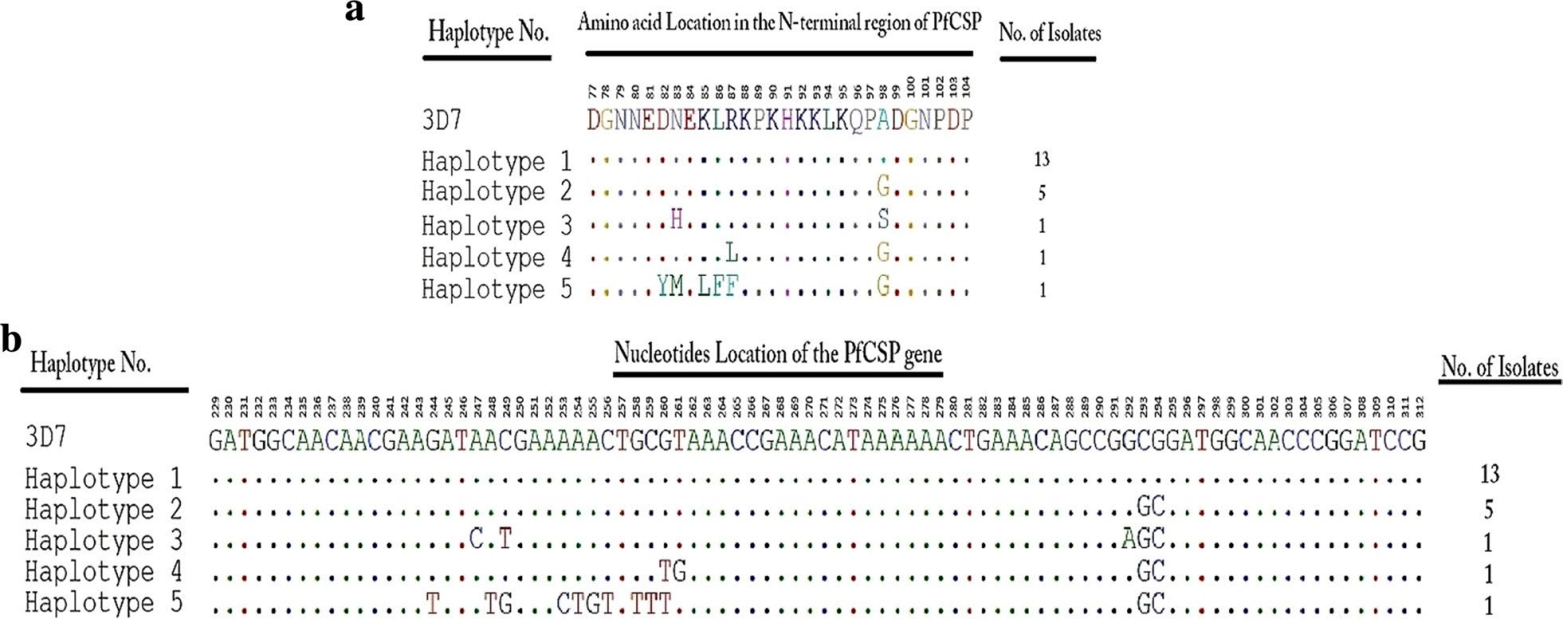
Number of Sudanese *pfcsp* N-terminal region haplotypes detected in comparison with the 3D7 reference strain. **a** Amino acids alignment of the N-terminal region. Showing the different haplotypes detected among the study samples compared with the reference strains; Haplotype 1 include: 3D7 (XM_001351086), NF54 (M22982.1), HB3 (AB121018.1), haplotype 2 include: 7G8 (AB121015.1), Dd2 (AB121017.1), MAD20 (AB121020.1), RO33 (AB121021.1), Wellcome strain (M15505.1). Haplotype 3–5 are the different haplotypes detected among the study samples. KLKQP motif is conserved through all the study samples. **b** Nucleotides alignment of the 5′ to 3′ end of the Sudanese *pfcsp* gene with the reference strains

The analysis of the amino acids of the N-terminal region of the Sudanese PfCSP in comparison with the reference strains showed five different haplotypes (H). Two haplotypes were common; H1 and H2, while each of H3, H4, and H5 were found in 3 distinct isolates. H1 consisted of 3D7 (XM_001351086), NF54 (M22982.1), HB3 (AB121018.1) and 13 isolates of the Sudanese PfCSP. Whereas, H2 included 7G8 (AB121015.1), Dd2 (AB121017.1), MAD20 (AB121020.1), RO33 (AB121021.1), Wellcome strain (M15505.1) and 5 of the Sudanese isolates. Interestingly, H3, H4, and H5 have consisted of only one isolate of the Sudanese isolates for each haplotype. The KLKQP motif responsible for the sporozoites entry and invasion of hepatic cells was highly conserved among all the studied samples. Also, all polymorphic sites in the N-terminal region were conservative polymorphisms, in H2 the only polymorphic site was A98G, while N83H and A98S polymorphisms were present in H3, whereas R87L was found in H4. Meanwhile, several polymorphic sites including D82Y, N83M, K85L, L86F, and R87F were found in H5 (Fig. 3a: Amino acids alignment of the N-terminal region). Haplotype diversity (Hd) was 0.594 ± 0.065 with a nucleotide diversity (Pi) of 0.01654 and variance of haplotype diversity of 0.00417. Also, the average number of pairwise nucleotide differences (k) was 1.389. Fu and Li’s D* test statistic value was − 2.70818 (P < 0.05), Fu and Li’s F* test statistic value was − 2.83907 (P < 0.05). The number of polymorphic (segregating) sites (S) detected in the *pfcsp* gene were 12, suggesting the number of polymorphic sites might tend to be more if big sample size has been used (Fig. 3b: nucleotides alignment of the 5′ to 3′ end of the *pfcsp* gene).

**Fig. 4:**
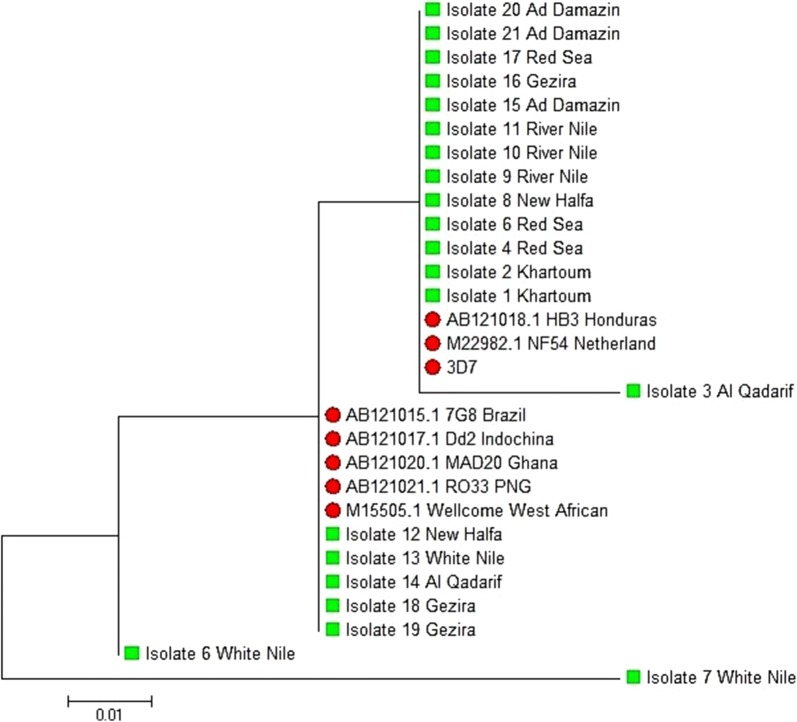
Phylogenetic tree of the N-terminal region of the Sudanese *pfcsp* with the worldwide reference strains. Phylogenetic tree based on the maximum likelihood method using Jukes and Cantor model for 8 *pfcsp* gene of the reference strains (marked with red circle), and 21 Sudanese isolates (marked with green box) labeled with their corresponding region

The constructed phylogenetic tree based on the maximum likelihood method using Jukes and Cantor model to describe the nucleotides substitution pattern with the reference strains showed that most of the Sudanese *pfcsp* N-terminal region sequences were firmly related to the 3D7, NF54 and HB3 reference strains. Only 2 isolates showed divergence from the reference strains (Fig. 4).

### Sequence analysis of the global PfCSP N-terminal region

**Fig. 5:**
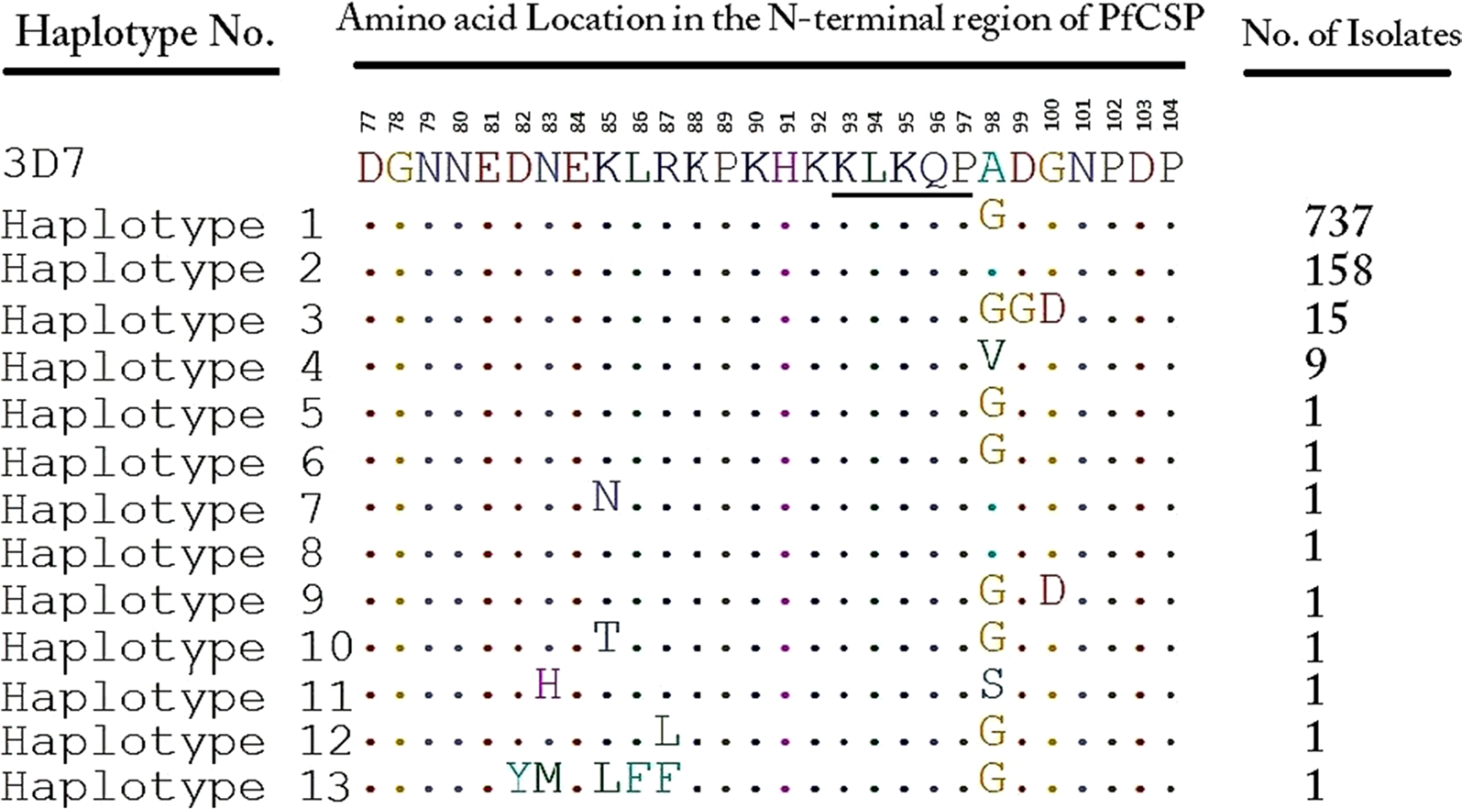
Amino acids alignment of the global *pfcsp* N-terminal region. A total of 13 different haplotypes were detected. Most of the study isolates were similar to Haplotype 1 and Haplotype 2. Haplotypes 11, 12 and 13 are the different haplotypes detected among the study isolates. Underlined KLKQP motif is conserved through all the global *pfcsp* N-terminal regions sequences and the Sudanese *pfcsp*

**Fig. 6:**
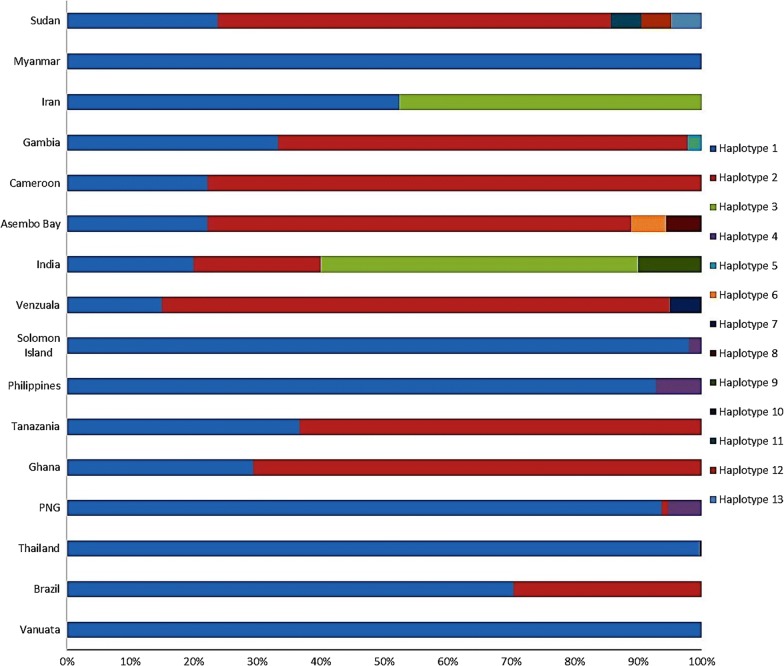
Frequency comparison of N-terminal region of global PfCSP from different geographic regions. Frequency distribution of the 13 haplotypes detected in the global PfCSP N-terminal region and the Sudanese PfCSP. Haplotype 2 had the highest frequency among the Sudanese isolates

Analysis of the global N-terminal regions of 927 published *pfcsp* sequences (see Additional file 3), and 21 sequences of the current study showed that this region is relatively well-conserved. Amino acid polymorphisms identified in the N-terminal region of PfCSP were present at eight positions (D82Y, N83H/M, K85L/T/N, L86F, R87L/F, A98G/V/S, D99G and G100D). Also, no insertion at the PfCSP in the N-terminal region of Sudan isolates was identified, i.e. (NNGDNGREGKDEDKRDGNN). Figure 5 shows the amino acids alignment of the N-terminal region of global PfCSP N-terminal region. Only 13 haplotypes have been detected through analysing the amino acids of the global *pfcsp*. H1 encompassed the highest frequency followed by H2 with a frequency of 79.4% and 17.0%, respectively. The frequencies of the remainder haplotypes were 1.6% and 1.0% for H3 and H4, respectively, and 0.1% for each H5, H6, H7, H8, H9, H10, H11, H12, and H13 (Fig. 6). Hd was 0.340 ± 0.017 with a Pi of 0.00485 and variance of haplotype diversity of 0.00030. Also, k was 0.407. Fu and Li’s D* test statistic value was − 6.96713 (P < 0.02), Fu and Li’s F* test statistic value was − 5.99452 (P < 0.02). The number of segregating sites detected in the 5′ to 3′ end of the N-terminal region of the global PfCSP was 18. Tajima’s D value was − 1.98991 (P < 0.05). Fu and Li’s F and D test statistics and Tajima’s D test values indicate a role of negative balancing selection occurs in the N-terminal region.

## Discussion

The genetic diversity of the Sudanese *P. falciparum* has been comprehensively studied, with an unwavering focus on specific genetic markers that could discriminate the *P. falciparum* strains from each other [6–10, 38, 39]. This study aimed at investigating the genetic polymorphism of the Sudanese *P. falciparum* isolates based on the N-terminal region of PfCSP.

The Sudanese PfCSP has a well-conserved N-terminal region when compared to the worldwide *pfcsp* gene coinciding with populations from other geographical areas [14, 16, 30, 40–42]. This conservation is also corresponding to previous reports investigated the genetic diversity of PfCSP in a global scale study which showed low genetic diversity in the N-terminal region compared to the central repeat and the C-terminal regions [14, 16, 43, 44]. However, a few amino acid polymorphisms have been identified. Polymorphisms were consisting of A98G/S, N83H/M, R87L/F, D82Y, K85L, and L86F. Although, the A98G polymorphism was the only common identified polymorphism in the Sudanese isolates and the reference strains sequences of the N-terminal region of PfCSP, its frequency differed by country as its been indicated previously [16]. This divergence in frequency which also affects the genetic diversity in the N-terminal region could be owed to environmental pressures in terms of evading host immune response or evading drugs pressures such the case on the great Mekong sub-region or the Indian subcontinent [14, 16, 25]. Also, the diversity of circulating parasite strains in a specific region such as Sudan could implicate in the process of specific dominant strain in that region, and through time this could result in maintaining a specific strain that able to overcome not only the host immune response but also drug pressure [3, 5–10, 38]. Also, the ability of a monoclonal antibody to bind to the linear epitope in the N-terminal region has effectively neutralized the infection of the sporozoites in vivo; accordingly, besides the similarity in the PfCSP N-terminal region this region could provide a potential vaccine candidate against falciparum malaria infection [45]. Importantly, the N-terminal region of PfCSP is known to play a crucial role in sporozoites invasion of hepatic cells [42, 45–47]. The N-terminal region of the *pfcsp* gene that been studied in vivo, through the production of a monoclonal antibody interact to the T cell epitope showed a productive neutralizing activity of sporozoites infectivity and hindering the entry into the hepatocytes [48, 49].

Most amino acid polymorphisms identified in the N-terminal region of global PfCSP were located in the predicted Th1R T cell epitope region, indicating that this region is under host immune responses [14, 16]. Although, The N-terminal region of PfCSP has been primarily neglected also despite being a target of inhibitory antibodies and protective T cell responses it showed an important role in playing a potential vaccine target [42, 50–52].

Although some studies indicated a particular insertion occurred in the N-terminal region of the *pfcsp* gene [14, 16], none of the sequenced Sudanese isolates showed any insertions in the N-terminal region such as been described previously in Myanmar isolates; a 19 amino acids insertion (NNGDNGREGKDEDKRDGNN) which was found in the middle of the N-terminal region [16]. However, this could also be reflected in the sample size being studied. Larger sample size from other different regions and also the selected regions of this study might provide different results if this insertion occurs by chance in the Sudanese *pfcsp* gene. Despite that, no any reports investigated the role of insertions that had been found in the N-terminal region.

Natural selection analysis of Sudanese and global PfCSP N-terminal region suggests that this region is likely to be under adverse balancing selection, which generates genetic diversity in the Sudanese PfCSP population. The dN–dS values for Sudanese *pfcsp* were negative, implying that balancing selection might not act in this region to maintain genetic diversity. These results did suggest that Sudanese *pfcsp* is under a complicated influence of natural selection, in which positive purifying selection might have occurred in the population, depending on the specific geographical origin of the parasite [16]. As previously discussed, higher values of recombination events found in African PfCSP than in PfCSP from other geographical areas, suggesting that African PfCSP might allow for more opportunity for multi-allelic recombination [43]. Moreover, this might also be reflected in the Sudanese PfCSP, which may also be due to the high multi-clonal infection rate and active recombination in mosquitoes [14, 16].

As presented in this study, the genetic diversity of the Sudanese PfCSP N-terminal region could focus on this region when developing a universal PfCSP-based vaccine, effective in a variety of areas. Nonetheless, if it is challenging to develop an effective vaccine that works against global malaria parasite populations, the development of a regional vaccine that works in certain malaria transmission areas can also be considered. For example, considering that H1 and H2 are the most prevalent haplotypes of PfCSP in the Sudanese and global PfCSP populations, these haplotypes could be considered in designing a PfCSP-based vaccine to be used in the different Sudanese regions.

## Conclusion

Collectively, this study provides information on the genetic diversity of the N-terminal region of PfCSP in Sudan. The relatively low genetic polymorphism in the N-terminal region of Sudanese PfCSP supports the concept that this region could be an ideal module of a CSP-based vaccine. The high similarity with other African isolates could contribute in the deployment of the PfCSP-based vaccine RTS,S in Sudan.

## Supplementary information

**Additional file 1: Table S1.** Descriptive, clinical data of the patients and results of nested PCR genotyping. **Table S2.** Merozoite Surface Protein-1 (*msp1*) genotyping results.

**Additional file 2: Figure S1.** Results of PCR amplification of the *P. falciparum csp* gene. MM: Molecular marker of 100 bp. Well No. 1: Positive control 3D7, wells 2–5, 7 and 8: Positive samples for the *csp* gene (product length 1200 bp). Well No. 6: Negative control.

**Additional file 3.** The global N-terminal regions of the published *pfcsp* sequences listed by country of Isolation.

## Data Availability

The datasets used in this study are available from the corresponding author on a reasonable request. Sequences used or analysed in this study were not submitted into the NCBI database.

## Abbreviations

H: haplotype
Hd: haplotype diversity
k: average number of nucleotide differences
kDa: kilo dalton
PCR: polymerase chain reaction
*pfcsp*: circumsporozoite surface protein of *Plasmodium falciparum*
Pi: nucleotide diversity
S: segregating sites
π: nucleotide diversity
