# Supplementary material for: Genetic polymorphism of the N-terminal region in circumsporozoite surface protein of Plasmodium falciparum field isolates from Sudan
Source: Malar J. 2019 Oct 1;18:333. doi: 10.1186/s12936-019-2970-0 (PMC6771110; doi:10.1186/s12936-019-2970-0)
Supplement: Supplementary file 1 — Additional file 1: Table S1. Descriptive, clinical data of the patients and results of nested PCR genotyping. Table S2. Merozoite Surface Protein-1 (msp1) genotyping results. [file 12936_2019_2970_MOESM1_ESM.docx]

**Additional file 1:**

**Table S1:** Descriptive, clinical data of the patients and results of nested PCR genotyping.

| Location ^a^ | No. of patients | Age range ^b^ | Gender | | Hb g/dl | Parasitemia parasite/µl | Microscopic results | | Nested PCR results | | Malaria Phenotype ^c^ |
| --- | --- | --- | --- | --- | --- | --- | --- | --- | --- | --- | --- |
|  |  |  | male | female |  |  | Positive* | Negative | Positive* | Negative |  |
| KH | 5 | 15-27 | 3 | 2 | 8.8 - 13.2 | 400 - 4300 | 5 | 0 | 5 | 0 | Un-C |
| NH | 9 | 21-48 | 4 | 5 | 10.0 - 14.1 | 500 - 7800 | 9 | 0 | 9 | 0 | Un-C |
| RS | 6 | 11-23 | 2 | 4 | 9.6 - 13.9 | 500 - 2200 | 6 | 0 | 6 | 0 | Un-C |
| WN | 3 | 20-42 | 3 | 0 | 12.1 - 14.3 | 1200 - 4400 | 3 | 0 | 3 | 0 | Un-C |
| QD | 7 | 16-47 | 5 | 2 | 10.2 - 14.4 | 980 - 5400 | 7 | 0 | 7 | 0 | Un-C |
| GZ | 9 | 10-52 | 2 | 7 | 10.2 - 13.4 | 500 - 6400 | 9 | 0 | 9 | 0 | Un-C |
| RN | 4 | 21-44 | 1 | 3 | 11.0 - 13.1 | 520 - 4400 | 4 | 0 | 4 | 0 | Un-C |
| AD | 7 | 9-32 | 3 | 4 | 10.7 - 13.9 | 640 - 3200 | 7 | 0 | 7 | 0 | Un-C |

^a^ KH; Khartoum, NH; New Halfa, RS; Red Sea, WN; White Nile, QD; Al Qadarif, GZ; Gezira, RN; River Nile, AD; Ad Damazin.

^b^ Age range in years.

^c^ Un-C; Un-Complicated.

* All Positive isolates were *P. falciparum* parasite

| Location^*^ | No. of patients | MSP-1 genotyping | | | | | | Clonal infection | |
| --- | --- | --- | --- | --- | --- | --- | --- | --- | --- |
|  |  | MAD20 | K1 | RO33 | MAD20/K1/RO33 | MAD20/K1 | K1/RO33 | Single | Multiple |
| KH | 5 | 1 | 1 | 0 | 2 | 1 | 0 | 2 | 3 |
| NH | 9 | 2 | 0 | 0 | 1 | 6 | 0 | 2 | 7 |
| RS | 6 | 2 | 0 | 1 | 3 | 0 | 0 | 3 | 3 |
| WN | 3 | 2 | 1 | 0 | 0 | 0 | 0 | 3 | 0 |
| QD | 7 | 2 | 0 | 0 | 5 | 0 | 0 | 2 | 5 |
| GZ | 9 | 2 | 0 | 1 | 6 | 0 | 0 | 3 | 6 |
| RN | 4 | 3 | 0 | 0 | 1 | 0 | 0 | 3 | 1 |
| AD | 7 | 1 | 2 | 0 | 4 | 0 | 0 | 3 | 4 |

**Table S2:** Merozoite Surface Protein-1 (MSP-1) genotyping results.

* KH; Khartoum, NH; New Halfa, RS; Red Sea, WN; White Nile, QD; Al Qadarif, GZ; Gezira, RN; River Nile, AD; Ad Damazin.
