# Supplementary material for: Genetic polymorphism of the N-terminal region in circumsporozoite surface protein of Plasmodium falciparum field isolates from Sudan
Source: Malar J. 2019 Oct 1;18:333. doi: 10.1186/s12936-019-2970-0 (PMC6771110; doi:10.1186/s12936-019-2970-0)
Supplement: Supplementary file 2 — Additional file 2: Figure S1. Results of PCR amplification of the P. falciparum csp gene. MM: Molecular marker of 100 bp. Well No. 1: Positive control 3D7, wells 2–5, 7 and 8: Positive samples for the csp gene (product length 1200 bp). Well No. 6: Negative control. [file 12936_2019_2970_MOESM2_ESM.docx]

**Additional file 2:**


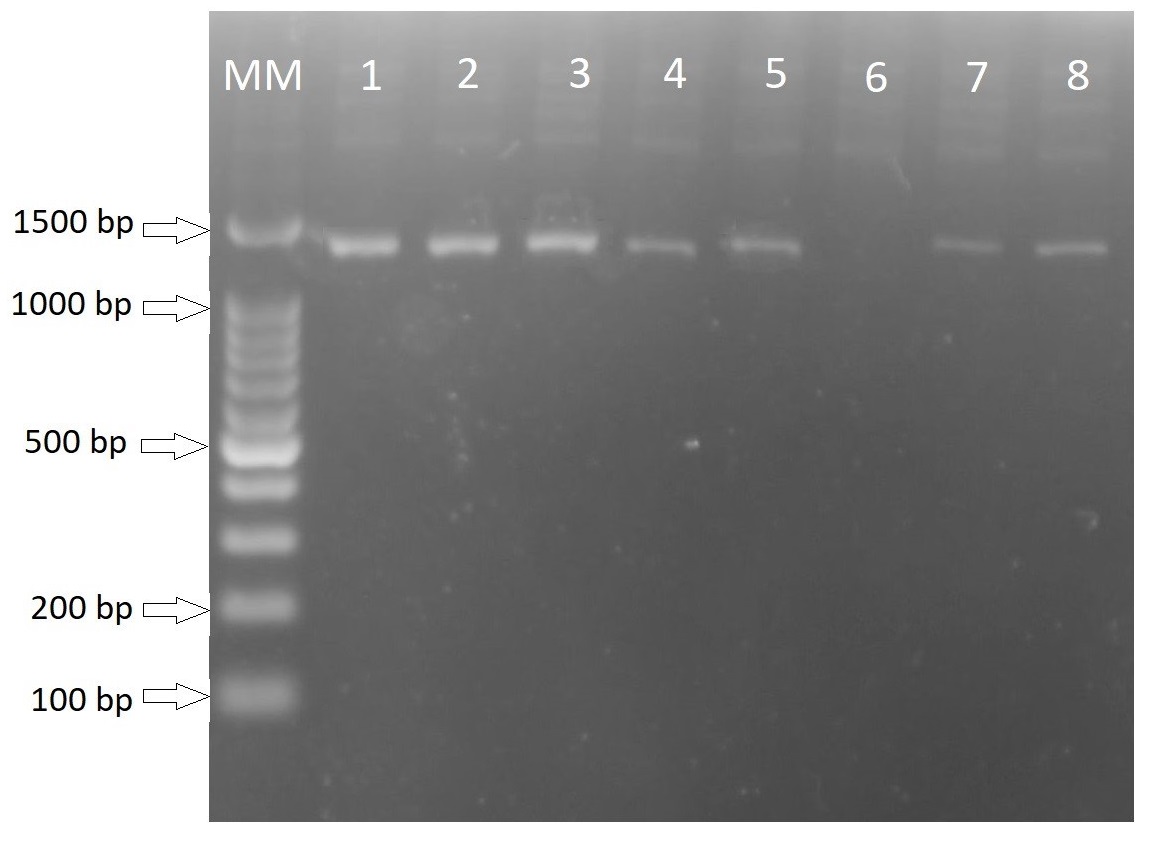


**Figure S1:** Results of PCR amplification of the *P. falciparum* *csp* gene. MM: Molecular marker of 100 bp. Well No. 1: Positive control 3D7, wells 2-5, 7 and 8: Positive samples for the *csp* gene (product length 1200 bp). Well No. 6: Negative control.
