# Supplementary material for: Genetic polymorphism of the N-terminal region in circumsporozoite surface protein of Plasmodium falciparum field isolates from Sudan
Source: Malar J. 2019 Oct 1;18:333. doi: 10.1186/s12936-019-2970-0 (PMC6771110; doi:10.1186/s12936-019-2970-0)
Supplement: Supplementary file 3 — Additional file 3. The global N-terminal regions of the published pfcsp sequences listed by country of Isolation. [file 12936_2019_2970_MOESM3_ESM.docx]

**Additional file 3:** The global N-terminal regions of the published *pfcsp* sequences listed by country of Isolation.

**Asembo Bay**

AF540444.1 AF540448.1 AF540449.1 AF540451.1 AF540452.1

AF540456.1 AF540457.1 AF540458.1 AF540462.1 AF540463.1

AF540464.1 AF540466.1 AF540468.1 AF540474.1 AF540475.1

AF540476.1 AF540477.1 AF540478.1

**Brazil**

AB121015.1 AB503152.1 AB503153.1 AB503154.1 AB503155.1

AB503156.1 AB503157.1 AB503158.1 AB503159.1 AB503160.1

AB503161.1 AB503162.1 AB503163.1 AB503164.1 AB503165.1

AB503166.1 AB503167.1 AB503168.1 AB503169.1 AB503170.1

AB503171.1 AB503172.1 AB503173.1 AB503174.1 AB503175.1

AB503176.1 AB503177.1 AB503178.1 AB503179.1 AB503180.1

AB503181.1 AB503182.1 AB503183.1 AB503184.1 AB503185.1

AB503186.1 AB503187.1 AB503188.1 AB503189.1 AB503190.1

AB503191.1 AB503192.1 AB503193.1 K02194.1

**Cameroon**

AF540445.1 AF540446.1 AF540447.1 AF540450.1 AF540453.1

AF540454.1 AF540455.1 AF540469.1 AF540481.1

**Gambia**

AF540441.1 AY878598.1 AY878599.1 AY878600.1 AY878601.1

AY878602.1 AY878603.1 AY878604.1 AY878605.1 AY878606.1

AY878607.1 AY878608.1 AY878609.1 AY878610.1 AY878611.1

AY878612.1 AY878613.1 AY878614.1 AY878615.1 AY878616.1

AY878617.1 AY878618.1 AY878619.1 AY878620.1 AY878621.1

AY878622.1 AY878623.1 AY878624.1 AY878625.1 AY878626.1

AY878627.1 AY878628.1 AY878629.1 AY878630.1 AY878631.1

AY878632.1 AY878633.1 AY878634.1 AY878635.1 AY878636.1

AY878637.1 AY878638.1 AY878639.1 AY878640.1 AY878641.1

**Ghana**

AB121021.1 AB502856.1 AB502857.1 AB502858.1 AB502859.1

AB502860.1 AB502861.1 AB502862.1 AB502863.1 AB502864.1

AB502865.1 AB502866.1 AB502867.1 AB502868.1 AB502869.1

AB502870.1 AB502871.1 AB502872.1 AB502873.1 AB502874.1

AB502875.1 AB502876.1 AB502877.1 AB502878.1 AB502879.1

AB502880.1 AB502881.1 AB502882.1 AB502883.1 AB502884.1

AB502885.1 AB502886.1 AB502887.1 AB502888.1

**India**

AF540442.1 AF540443.1 AF540473.1 AF540482.1 AF540483.1

AF540484.1 AF540485.1 AF540486.1 AF540487.1 AF540488.1

**Iran**

DQ521732.1 DQ521733.1 DQ521734.1 DQ521735.1 DQ521736.1

DQ521737.1 DQ521738.1 DQ521739.1 DQ521740.1 DQ521741.1

DQ521742.1 DQ521743.1 DQ521744.1 DQ521745.1 DQ521746.1

DQ521747.1 DQ521748.1 DQ521749.1 DQ521750.1 DQ521751.1

DQ521752.1

**Myanmar**

MF350670.1 MF350671.1 MF350672.1 MF350673.1 MF350674.1

MF350675.1 MF350676.1 MF350677.1 MF350678.1 MF350679.1

MF350680.1 MF350681.1 MF350682.1 MF350683.1 MF350684.1

MF350685.1 MF350686.1 MF350687.1 MF350688.1 MF350689.1

MF350690.1 MF350691.1 MF350692.1 MF350693.1 MF350694.1

MF350695.1 MF350696.1 MF350697.1 MF350698.1 MF350699.1

MF350700.1 MF350701.1 MF350702.1 MF350703.1 MF350704.1

MF350705.1 MF350706.1 MF350707.1 MF350708.1 MF350709.1

MF350710.1 MF350711.1 MF350712.1 MF350713.1 MF350714.1

MF350715.1 MF350716.1 MF350717.1 MF350718.1 MF350719.1

MF350720.1

**PNG**

AB121020.1 AB503007.1 AB503008.1 AB503009.1 AB503010.1

AB503011.1 AB503012.1 AB503013.1 AB503014.1 AB503015.1

AB503016.1 AB503017.1 AB503018.1 AB503019.1 AB503020.1

AB503021.1 AB503022.1 AB503023.1 AB503024.1 AB503025.1

AB503026.1 AB503027.1 AB503028.1 AB503029.1 AB503030.1

AB503031.1 AB503032.1 AB503033.1 AB503034.1 AB503035.1

AB503036.1 AB503037.1 AB503038.1 AB503039.1 AB503040.1

AB503041.1 AB503042.1 AB503043.1 AB503044.1 AB503045.1

AB503046.1 AB503047.1 AB503048.1 AB503049.1 AB503050.1

AB503051.1 AB503052.1 AB503053.1 AB503054.1 AB503055.1

AB503056.1 AB503057.1 AB503058.1 AB503059.1 AB503060.1

AB503061.1 AB503062.1 AB503063.1 AB503064.1 AB503065.1

AB503066.1 AB503067.1 AB503068.1 AB503069.1 AB503070.1

AB503071.1 AB503072.1 AB503073.1 AB503074.1 AB503075.1

AB503076.1 AB503077.1 AB503078.1 AB503079.1 AB503080.1

AB503081.1 AB503082.1 AB503083.1 AB503084.1 AB503085.1

AB503086.1 AB503087.1 AB503088.1 AB503089.1 AB503090.1

AB503091.1 AB503092.1 AB503093.1 AB503094.1 AB503095.1

AB503096.1 AB503097.1 AB503098.1 AB503099.1 AB503100.1

**Philippines**

AB502965.1 AB502966.1 AB502967.1 AB502968.1 AB502969.1

AB502970.1 AB502971.1 AB502972.1 AB502973.1 AB502974.1

AB502975.1 AB502976.1 AB502977.1 AB502978.1 AB502979.1

AB502980.1 AB502981.1 AB502982.1 AB502983.1 AB502984.1

AB502985.1 AB502986.1 AB502987.1 AB502988.1 AB502989.1

AB502990.1 AB502991.1 AB502992.1 AB502993.1 AB502994.1

AB502995.1 AB502996.1 AB502997.1 AB502998.1 AB502999.1

AB503000.1 AB503001.1 AB503002.1 AB503003.1 AB503004.1

AB503005.1 AB503006.1

**Solomon Islands**

AB503101.1 AB503102.1 AB503103.1 AB503104.1 AB503105.1

AB503106.1 AB503107.1 AB503108.1 AB503109.1 AB503110.1

AB503111.1 AB503112.1 AB503113.1 AB503114.1 AB503115.1

AB503116.1 AB503117.1 AB503118.1 AB503119.1 AB503120.1

AB503121.1 AB503122.1 AB503123.1 AB503124.1 AB503125.1

AB503126.1 AB503127.1 AB503128.1 AB503129.1 AB503130.1

AB503131.1 AB503132.1 AB503133.1 AB503134.1 AB503135.1

AB503136.1 AB503137.1 AB503138.1 AB503139.1 AB503140.1

AB503141.1 AB503142.1 AB503143.1 AB503144.1 AB503145.1

AB503146.1 AB503147.1 AB503148.1 AB503149.1 AB503150.1

AB503151.1

**Tanzania**

AB502796.1 AB502797.1 AB502798.1 AB502799.1 AB502800.1

AB502801.1 AB502802.1 AB502803.1 AB502804.1 AB502805.1

AB502806.1 AB502807.1 AB502808.1 AB502809.1 AB502810.1

AB502811.1 AB502812.1 AB502813.1 AB502814.1 AB502815.1

AB502816.1 AB502817.1 AB502818.1 AB502819.1 AB502820.1

AB502821.1 AB502822.1 AB502823.1 AB502824.1 AB502825.1

AB502826.1 AB502827.1 AB502828.1 AB502829.1 AB502830.1

AB502831.1 AB502832.1 AB502833.1 AB502834.1 AB502835.1

AB502836.1 AB502837.1 AB502838.1 AB502839.1 AB502840.1

AB502841.1 AB502842.1 AB502843.1 AB502844.1 AB502845.1

AB502846.1 AB502847.1 AB502848.1 AB502849.1 AB502850.1

AB502851.1 AB502852.1 AB502853.1 AB502854.1 AB502855.1

**Thailand**

AB121016.1 AB502889.1 AB502890.1 AB502891.1 AB502892.1

AB502893.1 AB502894.1 AB502895.1 AB502896.1 AB502897.1

AB502898.1 AB502899.1 AB502900.1 AB502901.1 AB502902.1

AB502903.1 AB502904.1 AB502905.1 AB502906.1 AB502907.1

AB502908.1 AB502909.1 AB502910.1 AB502911.1 AB502912.1

AB502913.1 AB502914.1 AB502915.1 AB502916.1 AB502917.1

AB502918.1 AB502919.1 AB502920.1 AB502921.1 AB502922.1

AB502923.1 AB502924.1 AB502925.1 AB502926.1 AB502927.1

AB502928.1 AB502929.1 AB502930.1 AB502931.1 AB502932.1

AB502933.1 AB502934.1 AB502935.1 AB502936.1 AB502937.1

AB502938.1 AB502939.1 AB502940.1 AB502941.1 AB502942.1

AB502943.1 AB502944.1 AB502945.1 AB502946.1 AB502947.1

AB502948.1 AB502949.1 AB502950.1 AB502951.1 AB502952.1

AB502953.1 AB502954.1 AB502955.1 AB502956.1 AB502957.1

AB502958.1 AB502959.1 AB502960.1 AB502961.1 AB502962.1

AB502963.1 AB502964.1 FJ232142.1 FJ232143.1 FJ232144.1

FJ232145.1 FJ232146.1 FJ232147.1 FJ232148.1 FJ232149.1

FJ232150.1 FJ232151.1 FJ232152.1 FJ232153.1 FJ232154.1

FJ232155.1 FJ232156.1 FJ232157.1 FJ232158.1 FJ232159.1

FJ232160.1 FJ232161.1 FJ232162.1 FJ232163.1 FJ232164.1

FJ232165.1 FJ232166.1 FJ232168.1 FJ232169.1 FJ232170.1

FJ232171.1 FJ232172.1 FJ232173.1 FJ232174.1 FJ232175.1

FJ232176.1 FJ232177.1 FJ232178.1 FJ232179.1 FJ232180.1

FJ232181.1 FJ232182.1 FJ232183.1 FJ232184.1 FJ232185.1

FJ232186.1 FJ232187.1 FJ232188.1 FJ232189.1 FJ232190.1

FJ232191.1 FJ232192.1 FJ232193.1 FJ232194.1 FJ232195.1

FJ232196.1 FJ232197.1 FJ232198.1 FJ232199.1 FJ232200.1

FJ232201.1 FJ232202.1 FJ232203.1 FJ232204.1 FJ232205.1

FJ232206.1 FJ232207.1 FJ232208.1 FJ232209.1 FJ232210.1

FJ232211.1 FJ232212.1 FJ232213.1 FJ232214.1 FJ232215.1

FJ232216.1 FJ232217.1 FJ232218.1 FJ232219.1 FJ232220.1

FJ232221.1 FJ232222.1 FJ232223.1 FJ232224.1 FJ232225.1

FJ232226.1 FJ232227.1 FJ232228.1 FJ232229.1 FJ232230.1

FJ232231.1 FJ232232.1 FJ232233.1 FJ232234.1 FJ232235.1

FJ232236.1 FJ232237.1 FJ232238.1 FJ232239.1 FJ232240.1

FJ232241.1 FJ232242.1 FJ232243.1 FJ232244.1 FJ232245.1

FJ232246.1 FJ232247.1 FJ232248.1 FJ232249.1 FJ232250.1

FJ232251.1 FJ232252.1 FJ232253.1 FJ232254.1 FJ232255.1

FJ232256.1 FJ232257.1 FJ232258.1 FJ232259.1 FJ232260.1

FJ232261.1 FJ232262.1 FJ232263.1 FJ232264.1 FJ232265.1

FJ232266.1 FJ232267.1 FJ232268.1 FJ232269.1 FJ232270.1

FJ232271.1 FJ232272.1 FJ232273.1 FJ232274.1 FJ232275.1

FJ232276.1 FJ232277.1 FJ232278.1 FJ232279.1 FJ232280.1

FJ232281.1 FJ232282.1 FJ232283.1 FJ232284.1 FJ232285.1

FJ232286.1 FJ232287.1 FJ232288.1 FJ232289.1 FJ232290.1

FJ232291.1 FJ232292.1 FJ232293.1 FJ232294.1 FJ232295.1

FJ232296.1 FJ232297.1 FJ232298.1 FJ232299.1 FJ232300.1

FJ232301.1 FJ232302.1 FJ232303.1 FJ232304.1 FJ232305.1

FJ232306.1 FJ232307.1 FJ232308.1 FJ232309.1 FJ232310.1

FJ232311.1 FJ232312.1 FJ232313.1 FJ232314.1 FJ232315.1

FJ232316.1 FJ232317.1 FJ232318.1 FJ232319.1 FJ232320.1

FJ232321.1 FJ232322.1 FJ232323.1 FJ232324.1 FJ232325.1

FJ232326.1 FJ232327.1 FJ232328.1 FJ232329.1 FJ232330.1

FJ232331.1 FJ232332.1 FJ232333.1 FJ232334.1 FJ232335.1

FJ232336.1 FJ232337.1 FJ232338.1 FJ232339.1 FJ232340.1

FJ232341.1 FJ232342.1 FJ232343.1 FJ232344.1 FJ232345.1

FJ232346.1 FJ232347.1 FJ232348.1 FJ232349.1 FJ232350.1

FJ232351.1 FJ232352.1 FJ232353.1 FJ232354.1 FJ232355.1

FJ232356.1 FJ232357.1 FJ232358.1 FJ232359.1 FJ232360.1

FJ232361.1 FJ232362.1 FJ232363.1 FJ232364.1 FJ232364.1

GQ890702.1 GQ890703.1 GQ890704.1 GQ890705.1 GQ890706.1

GQ890707.1 GQ890708.1 GQ890709.1 GQ890710.1 GQ890711.1

GQ890712.1 GQ890713.1 GQ890714.1 GQ890715.1 GQ890716.1

GQ890717.1 GQ890718.1 GQ890719.1 GQ890720.1 GQ890721.1

GQ890722.1 GQ890723.1 GQ890724.1 GQ890725.1 GQ890726.1

GQ890727.1 GQ890728.1 GQ890729.1 GQ890730.1 GQ890731.1

GQ890732.1 GQ890733.1 GQ890734.1 GQ890735.1 GQ890736.1

GQ890737.1 GQ890738.1 GQ890739.1 GQ890740.1 GQ890741.1

GQ890742.1 GQ890743.1 GQ890744.1 GQ890745.1 GQ890746.1

GQ890747.1 GQ890748.1 GQ890749.1 GQ890750.1 GQ890751.1

GQ890752.1 GQ890753.1 GQ890754.1 GQ890755.1 GQ890756.1

GQ890757.1 GQ890758.1 GQ890759.1 GQ890760.1 GQ890761.1

GQ890762.1 GQ890763.1 GQ890764.1 GQ890765.1 GQ890766.1

GQ890767.1 GQ890768.1 GQ890769.1 GQ890770.1 GQ890771.1

GQ890772.1 GQ890773.1 GQ890774.1 GQ890775.1 GQ890776.1

GQ890777.1 GQ890778.1 GQ890779.1 GQ890780.1 GQ890781.1

GQ890782.1 GQ890783.1 GQ890784.1 GQ890785.1 GQ890786.1

GQ890787.1 GQ890788.1 GQ890789.1 M19752.1 M83149.1

M83150.1 M83152.1 M83155.1 M83156.1 M83158.1

M83161.1 M83163.1 M83164.1 M83165.1 M83166.1

M83167.1 M83168.1 M83169.1 M83170.1 M83172.1

M83173.1 M83174.1

**Vanuatu**

AB116602.1 AB116603.1 AB116604.1 AB116605.1 AB116606.1

AB116607.1

**Venezuela**

AB503194.1 AB503195.1 AB503196.1 AB503197.1 AB503198.1

AB503199.1 AB503200.1 AB503201.1 AB503202.1 AB503203.1

AF540459.1 AF540460.1 AF540461.1 AF540465.1 AF540467.1

AF540470.1 AF540471.1 AF540472.1 AF540479.1 AF540480.1
